# Polyethylene glycol improves current methods for circulating extracellular vesicle-derived DNA isolation

**DOI:** 10.1186/s12967-019-1825-3

**Published:** 2019-03-11

**Authors:** N. García-Romero, R. Madurga, G. Rackov, I. Palacín-Aliana, R. Núñez-Torres, A. Asensi-Puig, J. Carrión-Navarro, S. Esteban-Rubio, H. Peinado, A. González-Neira, V. González-Rumayor, C. Belda-Iniesta, A. Ayuso-Sacido

**Affiliations:** 1 Fundación de Investigación HM Hospitales, HM Hospitales, C/Oña 10, 28050 Madrid, Spain; 2 IMDEA Nanoscience, Madrid, Spain; 3 Spanish National Cancer Research Center (CNIO), 28029 Madrid, Spain; 4 Atrys Health, Barcelona, Spain; 5 Facultad de Medicina (IMMA), Universidad San Pablo-CEU, Madrid, Spain

**Keywords:** Extracellular vesicles, Liquid biopsy, Polyethylene glycol, ExoQuick^®^, PureExo^®^, Ultracentrifugation

## Abstract

**Background:**

Extracellular vesicles (EVs) are small membrane-bound vesicles which play an important role in cell-to-cell communication. Their molecular cargo analysis is presented as a new source for biomarker detection, and it might provide an alternative to traditional solid biopsies. However, the most effective approach for EV isolation is not yet well established.

**Results:**

Here, we study the efficiency of the most common EV isolation methods-ultracentrifugation, Polyethlyene glycol and two commercial kits, Exoquick^®^ and PureExo^®^. We isolated circulating EVs from the bloodstream of healthy donors, characterized the size and yield of EVs and analyzed their protein profiles and concentration. Moreover, we have used for the first time Digital-PCR to identify and detect specific gDNA sequences, which has several implications for diagnostic and monitoring many types of diseases.

**Conclusions:**

Our findings present Polyethylene glycol precipitation as the most feasible and less cost-consuming EV isolation technique.

## Background

EVs are small cell-derived particles which play an important role in cell communication and are present in all body fluids [1]. At least three different groups of EVs can be defined based on their biogenesis and size: apoptotic bodies, shedding microvesicles and exosomes. They all contain diverse cellular molecules such as nucleic acids (DNA, RNA, miRNA), proteins, growth factors and lipids, and protect them from degradation [2]. In the last years, the interest in studying EV cargo has increased significantly, mainly due to its potential use as a biomarker source in liquid biopsy [3].

While solid biopsies are often unavailable or have small size [4], the major advantage of analyzing the molecular cargo of EVs in blood or urine is that these samples are obtained by prompt and minimally invasive procedures, which are suitable in a clinical setting. Furthermore, EV content represents the intratumor heterogeneity more faithfully, and provides a source of diagnostic and prognostic biomarkers that can be used to complement the patients’ data obtained by classical techniques [5].

Despite gaining tremendous scientific and clinical attention in the past decade, there is still no consensus regarding the most effective approach for EV isolation and their subsequent quantification and characterization. The original and most commonly used protocol for total EV isolation involves differential centrifugation and ultracentrifugation (UC), which can be followed by an UC on a sucrose or iodixanol gradient if extra pure exosome preparations are required [6, 7]. However, these laborious and time-consuming methods depend upon the utilization of specific and expensive equipment, which hinders EV clinical application [8]. Also, several parameters such as rotor type, viscosity of the sample and centrifugation time [9], need to be tightly controlled in order to standardize UC-based EV isolation across the labs. These disadvantages may be overcome by other techniques that separate EVs based on their size such as ultrafiltration, dialysis and size exclusion chromatography, such as gel-filtration [10–14]. These methods increase the purity and membrane integrity of obtained EVs, although usually render low recovery rates [15]. The immunoaffinity isolation uses the antibodies directed against EV surface proteins to obtain specific EV types. Together with the use of microfluidic devices [16], these systems seem promising for some applications, such as proteomic EV analysis, although they represent expensive alternatives that are not intended for purification of large amounts of EVs [17]. In addition, immunoisolation selects only the positive EVs for a chosen marker, which may not reflect the population as a whole. Finally, precipitation solutions represent an easy and fast approach for EV isolation which is mostly exploited by commercial kits [18]. While these kits are expensive, EV aggregates can be formed by a simple addition of polymers such as polyethylene glycol (PEG) and precipitated by a low-speed centrifugation [19].

Many efforts have been made to compare the efficiency of different EV isolation methods [20–24]. However, it still remains a challenge to decrease the inter/intra laboratory variability and define the optimal isolation method and starting volumes needed for different downstream applications [25]. In addition, pre-analytical conditions, such as sample processing and storage, may influence EV analysis and need to be fully standardized prior to clinical application [26].

**Fig. 1:**
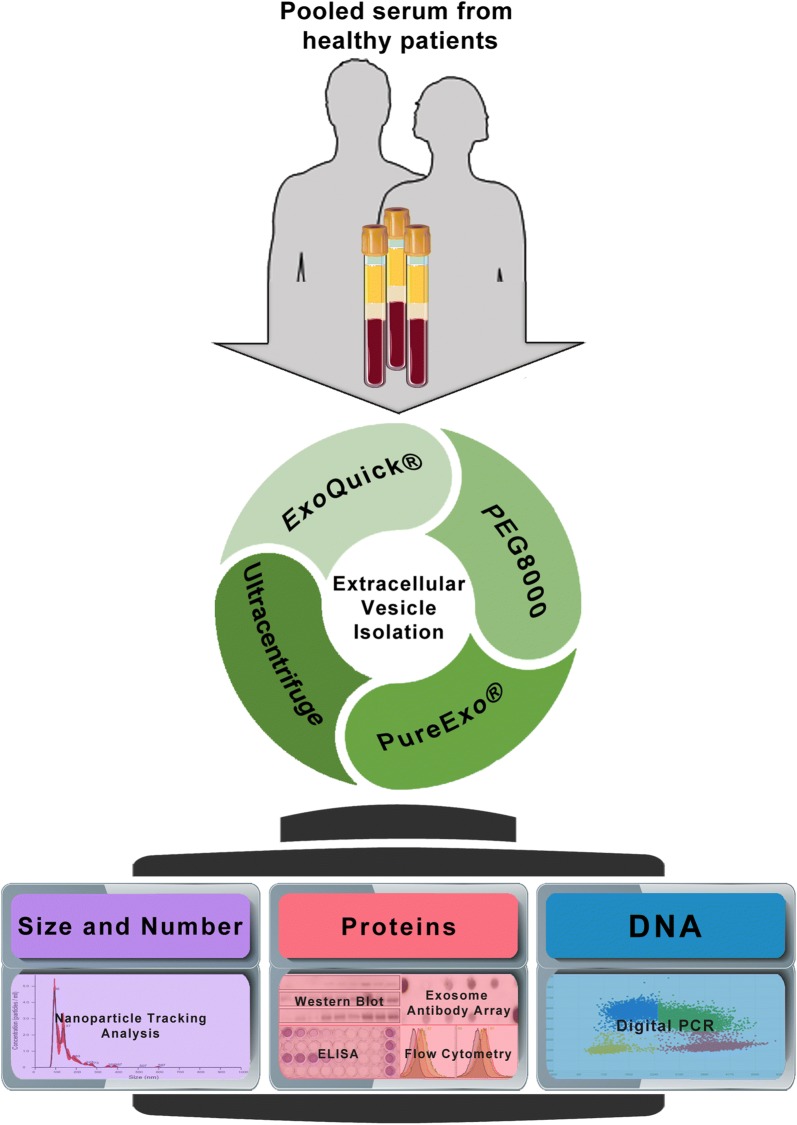
Experimental procedure flowchart

The aim of this article is to provide a comprehensive analysis covering the advantages and disadvantages of four EV isolation methods in order to define the most suitable method for DNA biomarkers detection in serum. We isolated EVs from different starting volumes of blood serum from healthy donors using four different techniques: UC, PEG precipitation, and two commercial kits (ExoQuick^®^ and PureExo^®^). We characterized the size and yield of enriched EVs using Nanoparticle Tracking Analysis (NTA), then determined their protein concentration, and finally analyzed their protein content using western blot, flow cytometry, ELISA and an antibody array (Fig. 1). Our data showed that PEG yields higher amounts of total EVs compared with the other methods tested. In addition, the EVs obtained by PEG had the highest DNA content, as we demonstrated by digital PCR (dPCR). Overall, our data suggest PEG as the most feasible and cost-effective isolation method, providing high yields of EVs suitable for DNA biomarker detection.

## Results

### PEG precipitation method enriches number of total isolated particles

The number of particles we have isolated was quantified using different techniques and three input serum volumes. The highest recovery values were obtained with PEG and Exoquick^®^ methods.

**Fig. 2:**
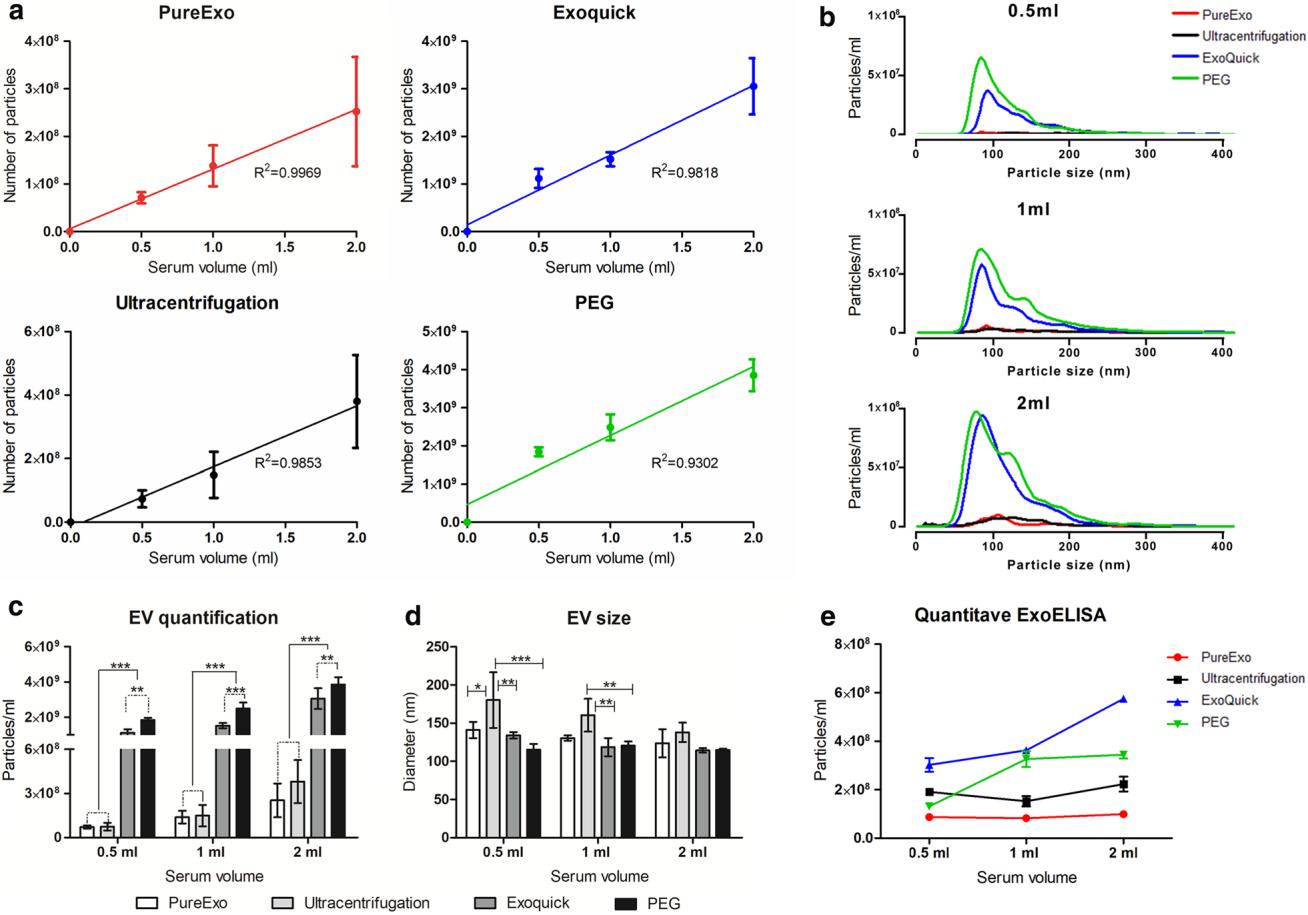
Particles size and number distribution. Correlation between the number of particles isolated and the serum volume (**a**). The NTA size distribution shows that PEG enriched a higher number of total EVs in 0.5, 1 and 2 ml (**b**). Total number of particles per ml quantification (**c**). EV size distribution (nm) (**d**). Quantitative ExoELISA assay of tetraspanin CD9. Data are shown as particles per ml (**e**). *p ≤ 0.05, **p ≤ 0.01, ***p ≤ 0.001. Data shown as mean ± S.D. Experiments were performed in duplicate and repeated three times

As a first approach, we evaluated the total number of particles obtained per ml and effectively proved that the number of isolated particles depends on the amount of starting material used. We observed a linear correlation with a strong relationship between the number of particles and the serum volume (Fig. 2a). Afterwards, particles recovery and size distribution were analyzed for each respective isolation method (Fig. 2b). The lowest number of particles (7.1 × 10^7^) was observed using 0.5 ml of input serum and PureExo^®^ method. In contrast, the highest yield of particles per ml (3.85 x 10^9^) was obtained using 2 ml of input serum and PEG. Surprisingly, we noticed that the UC method shows a similar number of particles per ml to PureExo^®^ in all the volumes analyzed, being significantly lower than the values observed for Exoquick^®^ and PEG (p < 0.001). These values were two orders of magnitude higher when we used 0.5 ml and were more than 10 times higher in 1 and 2 ml. Furthermore, the recovery rate of particles obtained using PEG was significantly higher than Exoquick^®^ (p < 0.01) (Fig. 2c).

To further calculate the average size of isolated particles, NTA diameter measurements were analyzed. As shown in Fig. 2d, the isolation method affects the size distribution profiles. These results indicated that particles isolated by UC have the largest diameter in all the cases studied, being significantly higher than the mean obtained by the other three methods for the starting volume of 0.5 ml, whereas in the case of 1 ml, significant differences were observed only among Exoquick^®^ and PEG (p < 0.01).

Moreover, the EV concentration was assayed and quantified by using the ExoELISA kit, which is based on the presence of the CD9 tetraspanin. The highest value was obtained with Exoquick^®^ and a 5.8-fold lower recovery was detected with 2 ml using the PureExo^®^ kit (Fig. 2e).

### PEG and Exoquick^®^ show the highest protein quantity

**Fig. 3:**
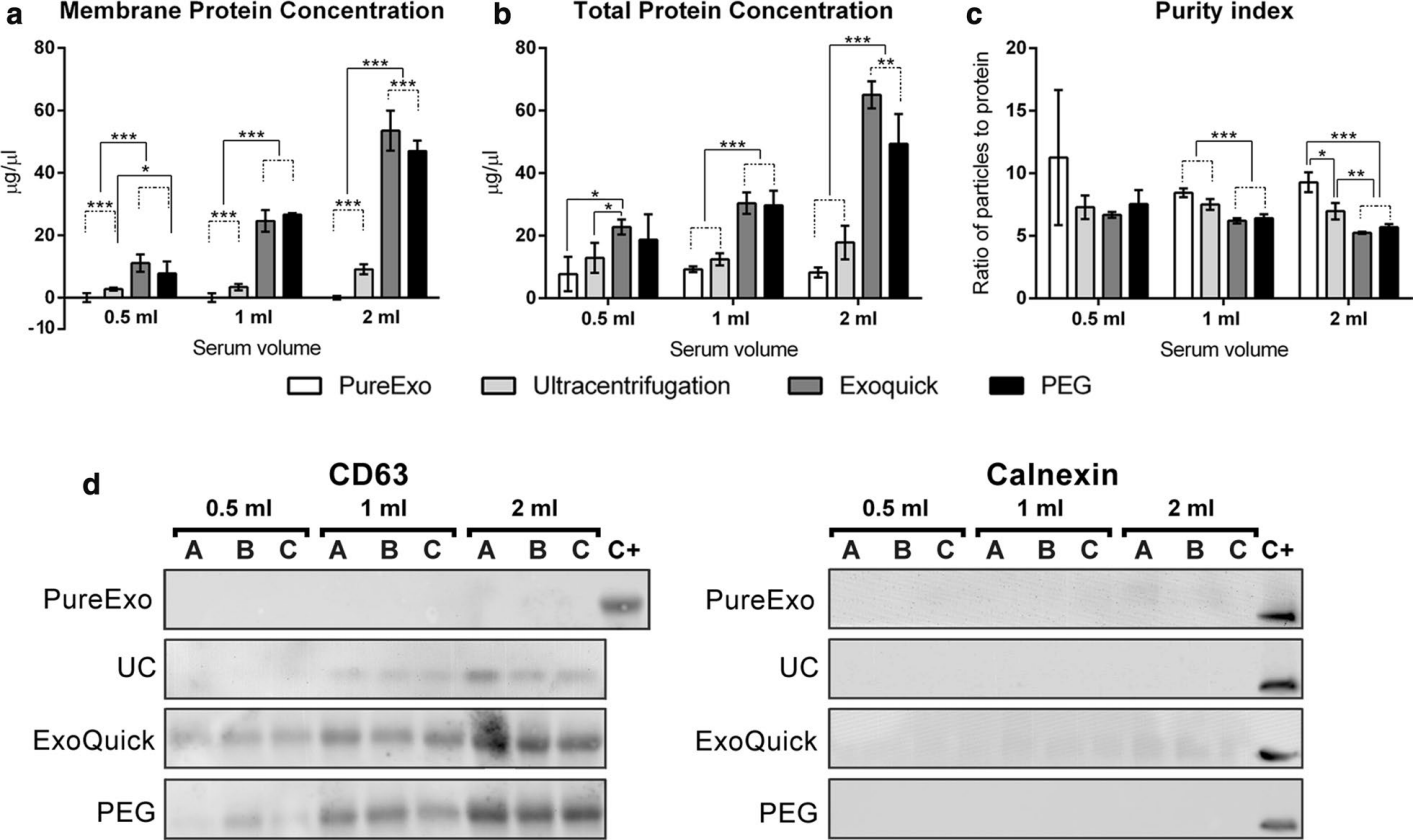
Enrichment of EV markers. Membrane protein quantification (**a**). Total protein quantification (**b**). Comparison of EV Purity index (**c**). Representative western blot showing CD63 protein levels and no signal of endoplasmic reticulum (Calnexin) (**d**)

To examine the efficiency of these four EV isolation methods, we have also compared the protein concentration. For this issue, we next quantified the amount of protein in EV membrane and observed similar values in EVs isolated by the commercial kit Exoquick^®^ and PEG except for 2 ml of starting serum, in which the concentration measures were higher in EVs isolated by Exoquick^®^ (p < 0.001). When using PureExo^®^ there were significant differences in all cases (p < 0.001), since proteins were only detected for the starting volumes of 2 ml (1.75 µg/µl), but not in the other volumes. Lower protein concentration (p < 0.001) was detected between UC and the group composed by Exoquick^®^ and PEG in all the volumes studied except in the samples obtained from UC and PEG with 0.5 ml of serum (p < 0.05) (Fig. 3a).

After EV lysis, we quantified the total protein extracted from EVs obtained by the four isolation methods. Consistent with previous membrane quantification results, we observed lower yields of proteins using PureExo^®^ and UC as compared to Exoquick^®^ and PEG (p < 0.001) in 1- and 2-ml samples. Additionally, using 0.5 ml of serum, only significant differences were shown in Exoquick^®^ when samples were compared to UC and PureExo^®^ (p < 0.05) (Fig. 3b). Since the relationship between the number of particles and the proteins isolated is an important factor to consider, we examined this ratio in our data. As shown in Fig. 3c, we observed the highest purity with PureExo^®^ followed by UC isolation method, suggesting the presence of less protein contaminants with these methods. However, the lower values were observed with Exoquick^®^ and PEG precipitation method.

### Enriched population contains EV proteins and no cellular contamination

**Fig. 4:**
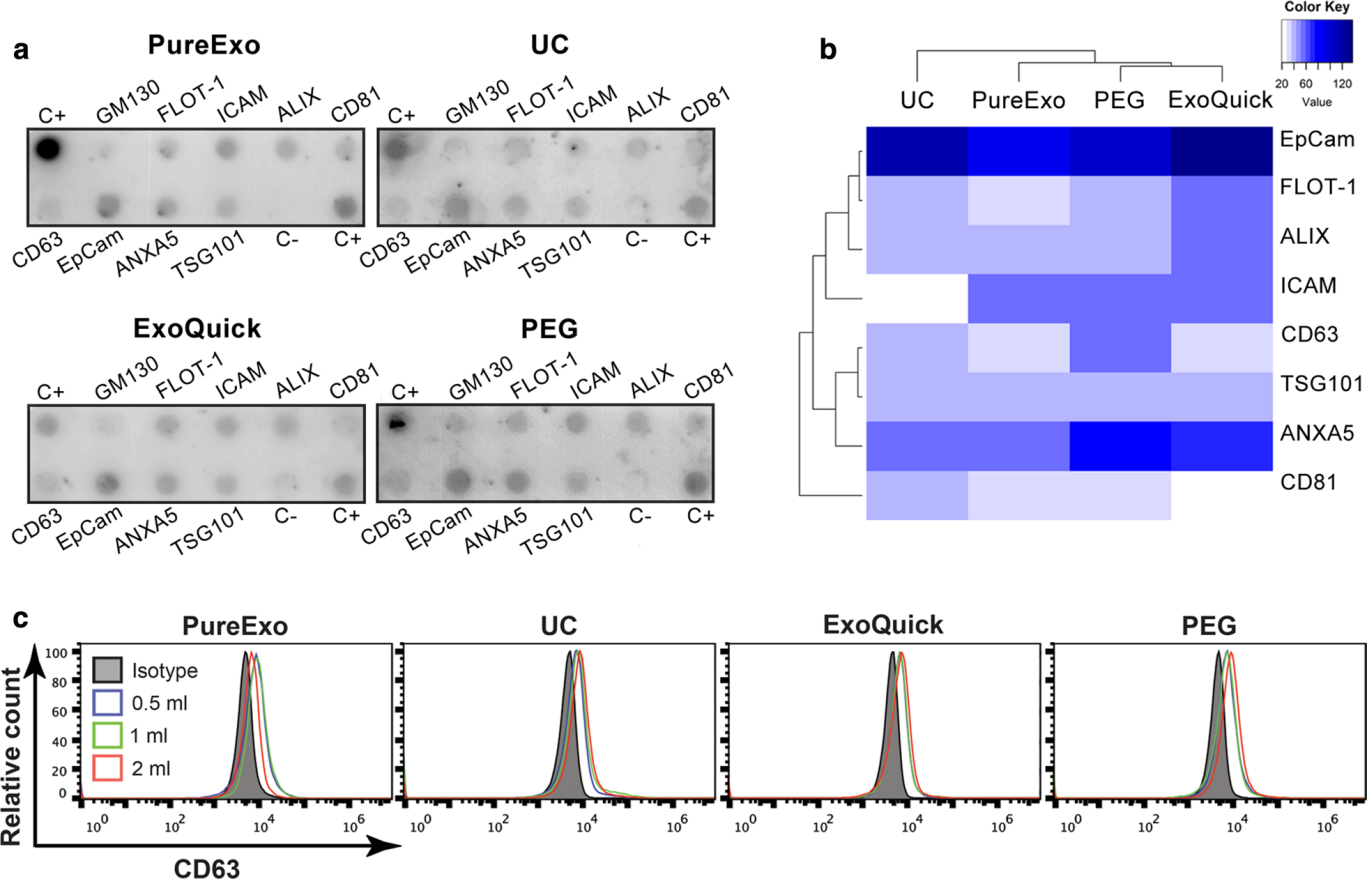
Exosome protein expression array (**a**). Heatmap of EV markers protein expression (white, none or very low expression; light blue, low expression; dark blue, high expression) (**b**). Analysis of CD63 expression by flow cytometry (**c**). Data are presented as mean ± S.D of three independent experiments, each performed in duplicate

To verify that our samples are enriched in typical EV markers, we examined the expression of the transmembrane tetraspanin protein CD63, by western blot. We detected its expression in all the volumes studied in PEG and Exoquick^®^. However, in the case of samples isolated by UC, CD63 expression was only observed for the starting serum volumes of 1 and 2 ml, and no signal appeared in PureExo^®^ samples (Fig. 3d). For a better characterization of the isolated EVs, we checked the protein expression profile based on a pre-printed antibody array encompassing 8 EVs markers (FLOT-1, ICAM, ALIX, CD81, CD63, EpCam, ANXA5 and TSG101) (Fig. 4a). The relative expression of these markers is represented as a heatmap, and the four methods are classified in three different clusters based on their protein profile.

Figure 4b shows that PEG EV marker profiles were strongly correlated with the ones from Exoquick^®^. Both of them had some similarities to EVs isolated by PureExo^®^, however the UC profile is the least correlated method. Additionally, we demonstrated no cellular contamination in our samples, analyzing the endoplasmic reticulum marker calnexin by western blot, and the cis-Golgi marker, GM130, in the antibody array (Figs. 3d, 4a).

In order to validate the results described above, we performed a flow cytometry analysis of EV samples bound to aldehyde/sulfate latex beads. Our data confirmed the presence of CD63 on the surface of all EVs isolated (Fig. 4c).

### Isolation with PEG results in a higher DNA copy number

**Fig. 5:**
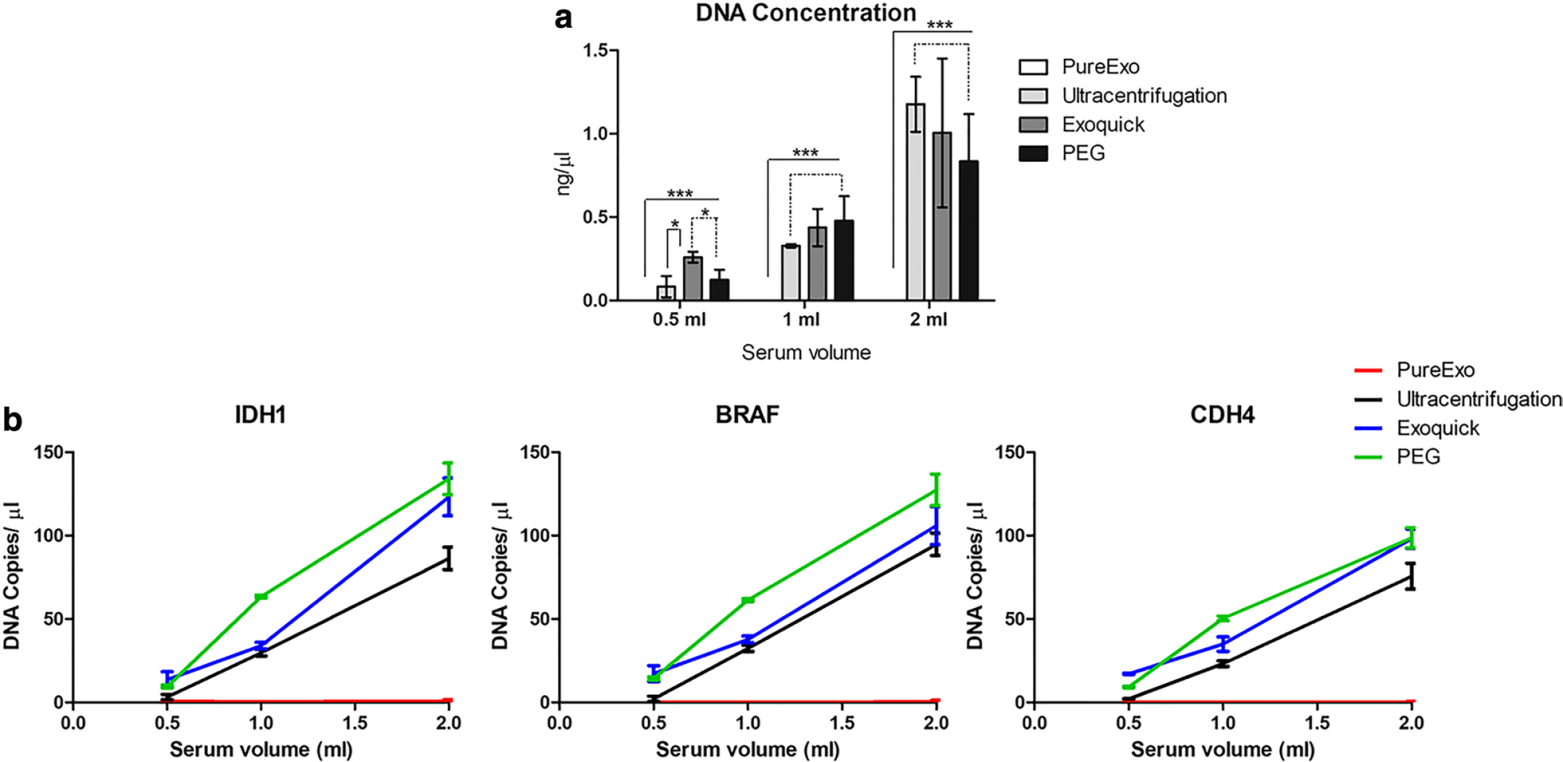
Genomic DNA quantification. Double-stranded DNA quantification using the Qubit Fluorometer. *p ≤ 0.05, ***p ≤ 0.001 (**a**). Digital PCR analysis for the absolute quantification (copies per µl) of three different probes (*IDH1*, *BRAF* and *CDH4*) (**b**). Error bars represent standard deviation

Our last set of experiments show that PEG yielded the highest DNA isolation efficiency. To measure the recovery rate of DNA obtained, we used two different approaches. Firstly, we quantified the dsDNA concentration with a fluorometric assay showing non-detectable values in samples isolated using PureExo^®^ and similar results among the other three methods used. Significantly greater values were observed among Exoquick^®^, PEG and UC using 0.5 ml of serum (p < 0.05) (Fig. 5a).

Secondly, a digital PCR was performed to demonstrate EV clinical utility. We analyzed the number of copies per µl of three genes (*IDH1*, *BRAF* and *CDH4*). Remarkably, similar values were obtained in all three probes analyzed, showing the high repeatability of the technique. According to the previous experiments, the highest DNA yield recovery was obtained after PEG isolation method, with a maximum concentration of 134.14 ± 9.43 copies of DNA per µl in the *IDH1* gene, followed by the Exoquick^®^ method and UC. Less than one copy per µl was observed in the samples obtained by the commercial kit PureExo^®^ (Fig. 5b).

In summary, we have made a comparison among the four EV isolation methods, in which, PEG is presented as the most feasible EV isolation method, having the highest global score, followed by the high-costEV precipitation technique -Exoquick^®^-. Surprisingly, the most expensive commercial kit, PureExo^®^, is shown as the least favourable choice. Although we have obtained the purest EV population, the number of particles, proteins and gDNA have displayed the lowest recovery efficiency (Table 1).

**Table 1:** Comparison among the different EV isolation methods with 2 ml serum

EVs isolation method	Number of particles score	Size score	Total protein score	Purity score	DNA copies score	Price
PureExo^®^	1	3	1	4	1	4
Ultracentrifugation	2	4	1	3	2	1
Exoquick^®^	3	2	4	2	3	3
PEG	4	2	3	2	4	1

Score from 1 (the lowest) to 4 (the highest)

## Discussion

Identification of molecular biomarkers in liquid biopsies from cancer patients is becoming quite a useful technique. While some tumors like colorectal, breast or lung release a considerable amount of macromolecules to the bloodstream, others display important limitations due in part to their grade, location or size [27, 28]. Furthermore, new approaches in the modern genomic medicine that require higher DNA input material are emerging, such as the target deep sequencing and the whole exome sequencing [29, 30]. Their analysis could help in tumor genotyping potentially leading to a successful screening, an early diagnosis, response and follow up [5, 29–35].

Thus, continuous refinement of isolation techniques in order to improve sensitivity and specificity of biomarker detection is essential. With this challenge in mind, here we obtained EVs from serum of healthy donors by UC, two commercial kits based on polymer-based precipitation reagents and PEG and compared the potential of EV gDNA as a biomarker source. Since there is not a well-established technique to quantify and identify EVs [36], we adopted several approaches for their characterization.

Firstly, we have evaluated the number of isolated particles by NTA. We obtained the highest recovery of particles using PEG, following the same trend published by Tang and colleagues [37, 24]. Although PEG has recently been proposed as a method for EV enrichment [19], there is a lack of comparative studies in the literature. Our results point out that PEG is a real alternative to the expensive commercial precipitation reagents and the arduous UC method for EV enrichment. Furthermore, it has recently been presented as a scalable method for different volumes in other kind of samples as supernatant of cell and tissue cultures [38]. Consistent with the fact that the time and g-force used for UC method precipitates EVs of all sizes, this technique yielded EVs of greater diameter. Furthermore, organic solvents present in other three methods encourage the precipitation of smaller size EVs, and not ABs, since their membrane composition is different [39]. Our findings are consistent with those observed in other serum samples [22].

Other indirect approaches to estimate the number of EVs are to measure the protein concentration and to perform an antibody-based quantification (ExoELISA) [40]. We, therefore, obtained the greatest protein recovery using Exoquick^®^. These findings are not consistent with the EV numbers observed by NTA, in which the highest values were achieved by PEG. This might be due to the fact that this commercial kit may sediment contaminating proteins together with EVs and therefore overestimate the protein concentration [41, 42]. This has been reflected in the purity index, in which we could observe a slightly higher value in PEG than Exoquick^®^. However, both isolation methods appear to precipitate more proteins from the serum than UC and PureExo^®^, without affecting the total DNA recovery. We further confirmed that all samples -even EVs obtained by PureExo^®^- present typical EV protein markers, such as CD63, ICAM, ALIX, CD81 and ANXA5, among others. Distinct protein profiles were observed in EVs obtained by UC. This difference is consistent with the data observed in size distribution, as bigger EVs have different surface markers due to their different biogenesis and release mechanisms [43]. Furthermore, we determined our population was free of cellular contamination since there was no signal for organelle markers Calnexin and GM130 [44]. Additionally, we confirmed by flow cytometry that CD63 positive population values were similar to those values published in healthy donors [31].

Finally, the development of optimal techniques that provide enough genetic material is one of the main problems to overcome in liquid biopsies [45]. In this work, we compare DNA obtained from our samples after all four isolation methods and apply (for the first time to our knowledge) digital PCR to evaluate gDNA recovery in EVs.

As of yet, 0.5 ml of serum is enough to isolate sufficient EV DNA to perform d-PCR, which facilitates sample collection and processing. Surprisingly, although we have reported lower yields of EVs in samples isolated by UC, we have observed similar DNA values as PEG and Exoquick^®^. This might be explained by the presence of ABs carrying more DNA [46]. This is an important issue in this field as the genetic material comes from dying cells [47], thus it is not yet known if including them in a liquid biopsy is a good approach for biomarkers research [48]. Furthermore, PEG appears to be a reasonable alternative to traditional methods and commercial kits, being cheaper, more efficient and less time consuming than UC. As we have selected three genes which carry driver mutations relevant in several solid tumors, such as ovarian, lung, breast, skin and brain cancer [49–51] the higher gDNA values obtained by PEG present this method as a good approach to identify specific sequences to be used as prognostic, diagnostic or follow up biomarkers. Thus, it could help overcome the problems described in other methods and can be used for monitoring patients [52]. Although we have previously demonstrated the presence of gDNA inside EVs [27], it is worth mentioning that in the present report the main objective was to optimize the gDNA detection for biomarker studies, so the total gDNA obtained could be composed of EV and circulating gDNA [53], as no DNAse treatment has been used. Further investigations are needed in this field to address the possibility of using PEG for other downstream applications.

## Conclusions

We have successfully shown that PEG could stand as a robust method to isolate gDNA-EVs reducing costs and time and increasing gDNA yields. Our results could serve as a guide for EV DNA isolation standardization, providing a minimally invasive source of biomarkers in clinical practice. Our work also addresses the need to follow one specific isolation method depending on the research application, as different methods might account for different gDNA yields.

## Methods

### Samples

Pooled blood samples were obtained from 50 healthy donors from HM Hospitales, Madrid, Spain. Permission for their use was obtained from the ethical review board in HM Hospitales. These blood samples were left to clot at room temperature and serum was isolated and stored at − 80 °C. Three initial volumes of serum (0.5 ml, 1 ml and 2 ml) were compared.

### EV isolation

To remove contaminating cells and cellular debris serum samples were centrifuged at 3000*g* during 15 min. For the UC method, serum was ultracentrifuged at 117,000*g* for 90 min (Optima-LE 80 K ultracentrifuge, 55.2 Ti rotor; Beckman Coulter), the pellet was washed in Phosphate Buffered Saline 1X (PBS) (Invitrogen, 14040133), and ultracentrifuged again.

For the PEG method, 10% (w/v) PEG 8000 (Sigma Aldrich, 3015) was used to precipitate total EVs overnight (O/N) at 4 °C. Afterwards, the solution was centrifuged at 16,100*g* for 1 h.

Furthermore, two commercial kits (ExoQuick®; System Biosciences and PureExo®; 101Bio) were used according to the manufacturer’s instructions.

All centrifugations were performed at 4 °C. Total EVs were resuspended in 300 μl of PBS 1X and stored at − 80 °C until use. Experiments were performed in duplicate and repeated three times.

### Nanoparticle Tracking Analysis (NTA)

The samples were diluted 1:1000 in PBS 1X and particles size and concentration were analyzed using Nanosight NS500 instrument (Malvern Instrument). Videos were recorded 3 times for each sample, 60 s each and were repeated 3 times at a controlled temperature of 25  °C. Detection threshold was increased to 5 to reduce noise. The results were processed using the NTA 3.1. Software.

### Protein isolation and quantification

Protein concentration was determined using Bio-Rad Protein Assay according to the microassay procedure. The absorbance was then read at 595 nm in the Varioskan Flash (Thermo Fisher Scientific). Membrane protein concentration was measured directly. However, measurements of total EV protein levels were obtained after EV lysis using 50 µl of EVs previously resuspended in PBS 1X and 50 µl of Radioimmunoprecipitation buffer (RIPA), (20 mM Tris pH 7.5, 150 mM NaCl, 10 mM Ethylenediaminetetraacetic acid (EDTA), 1% Triton x-100, 5 mM NaF and protease/phosphatase inhibitor cocktail). Then, proteins were extracted by centrifugation at 13,200*g* during 20 min at 4 °C.

### Western blotting

Protein extracts (40 µl) were separated by 12% SDS-PAGE and transferred to nitrocellulose membranes. After blocking for 1 h with 10% Bovine Serum Albumin in Tween-Tris Buffered Saline 1X (T-TBS), membranes were incubated with primary antibody against CD63 (Abcam, ab8219, 1 µg/ml) O/N at 4 °C. After washing, membranes were incubated with a Peroxidase Horse anti- Mouse IgG, (1:2000, PI-2000, Vector) for 1 h at room temperature (RT). Detection was performed using ECL™ reagents according to the manufacturer’s guidelines (GE Healthcare).

### Flow cytometry analysis of CD63

EVs samples were adsorbed onto 4% w/v, 4-µm aldehyde/sulfate latex beads (Thermo Fisher Scientific) for 1 h at 4 °C. The ratio between the number of beads and EVs was kept constant (1:1) for all samples. The reaction was stopped by adding 100 mM glycine and washed at 800*g* with 1 ml of PBS 1X. Membrane-bound beads were incubated with mouse anti-CD63 (1:100, 556019, BD-Bioscience) for 1 h at RT and stained with a FITC-conjugated secondary antibody (1:200, R&D Systems) for 1 h at RT. After a final washing step, the samples were resuspended in 0.5 ml of PBS 1X and analyzed using the Attune Acoustic Focusing Cytometer (Thermo Fisher Scientific).

### EV quantification by CD9 ELISA

The concentration of EVs was indirectly determined by the amount of immunoreactive EV-associated CD9 (ExoELISA™, System Biosciences). EV samples (20 µl) were added to a 96-well plate and incubated at 37 °C O/N. Then, the plate was washed and incubated with a primary antibody (CD9) at RT for 1 h with agitation. After three washing steps, the samples were incubated with a secondary antibody (1:5000) and with super-sensitive TMB ELISA substrate. The absorbance was measured at 450 nm in the Varioskan. Final results were expressed as number of EVs per ml of serum.

### EV detection with Exo-Check™ antibody array

A membrane-based antibody array (Exo-Check™, System Biosciences) was used to detect 8 known EV markers (FLOT-1, ICAM, ALIX, CD81, CD63, EpCAM, ANXA5 and TSG101) following the manufacturer’s instructions. Briefly, 400 µg of EV protein was lysed and incubated O/N in the antibody membrane array. After washing steps, the membrane was incubated with a detection buffer and developed in a chemiluminescent imager.

Images were transformed to 8-bits using ImageJ. Then, the median value of gray intensity was measured for each spot. Data was normalized using the negative and positive controls of each array so that negative controls have a value of 0 and the positive controls a value of 100. We applied a hierarchical clustering to detect the relation between the normalized intensity of the EV markers in the different methods studied. The distance metric used was 1 minus the Pearson’s correlation coefficient.

### DNA extraction and quantification

200 μl of EVs were digested with 100 μl of lysis buffer [(50 mM NaCl (S5886, Sigma Aldrich), 5 mM EDTA (E9889, Sigma Aldrich), 5 mM Tris, pH 8.0 (T6066, Sigma Aldrich), 1% Sodium Dodecyl Sulfate (SDS, L3771, Sigma Aldrich), 20 mM Dithiothreitol (DTT, 43816, Sigma Aldrich) and 0.5 mg/ml of Proteinase K (P4850, Sigma Aldrich)] O/N at 56 °C. Then, DNA extraction was performed using the standard phenol: chloroform: isoamyl alcohol procedure. DNA concentration was determined using Qubit™ dsDNA High Sensitivity assay kit in a Qubit™ 3.0 fluorometer (Thermo Fisher Scientific) according to the manufacturer’s proceedings.

### Digital PCR

The digital PCR (d-PCR) was performed according to the reference protocol for standard and rare mutation using QuantStudio™ 3D Digital PCR System (Thermo Fisher Scientific). We combined EV DNA with nuclease-free H_2_O, QuantStudio™ 3D Digital PCR Master Mix, and the ready-to-order TaqMan Assays (20X) for rs6142884 (*CDH4*), rs28746 (*IDH1*) and rs113488022 (*BRAF*) (Thermo Fisher Scientific). Results were analyzed with QuantStudio™ 3D Analysis Suite Cloud Software (Thermo Fisher Scientific) and the mean number of copies per µl was calculated.

### Statistical analysis

Experimental results were statistically analyzed using ANOVA and the obtained *p* values were adjusted using Bonferroni method to counteract the multiple comparison problem. All the statistical analysis was performed using the software package GraphPad Prism 5.0 and values of α = 0.05 were used for hypothesis testing as statistically significant levels. The data in the graphs are presented as mean ± SD. *p ≤ 0.05; **p ≤ 0.01; ***p ≤ 0.001.

## Abbreviations

EVs: extracellular vesicles
UC: ultracentrifugation
PEG: polyethylene glycol
dPCR: digital PCR
PBS: Phosphate Buffered Saline
NTA: Nanoparticle Tracking Analysis
O/N: over night
RT: room temperature
